# Environmental Influence on the Occurrence of Multi-Organ Cystic Echinococcosis Infection in a Patient from Sardinia, Italy

**DOI:** 10.3390/diseases11030090

**Published:** 2023-06-27

**Authors:** Cinzia Santucciu, Paolo Albino Ferrari, Giulia Grimaldi, Alessandro Murenu, Sonia Nemolato, Piero Bonelli, Giovanna Masala, Giuseppe Salvatore Porcu, Roberto Cherchi

**Affiliations:** 1 WOAH and NRL for Echinococcosis, Animal Health, IZS della Sardegna, 07100 Sassari, Italy; 2 Division of Thoracic Surgery, Azienda di Rilievo Nazionale ed Alta Specializzazione “G. Brotzu”, 09121 Cagliari, Italy; paolo.a.ferrari@aob.it (P.A.F.); roberto.cherchi@aob.it (R.C.); 3 Department of Pathology, Azienda di Rilievo Nazionale ed Alta Specializzazione “G. Brotzu”, 09121 Cagliari, Italy; sonia.nemolato@aob.it (S.N.);

**Keywords:** clinic diagnosis, cystic echinococcosis, *Echinococcus granulosus s.l.*, rare clinical case, thoracic surgery

## Abstract

An uncommon clinical case of an adult woman who was referred to the hospital with severe symptoms attributable to cystic echinococcosis (CE) is described in this report. According to a questionnaire, the subject was exposed to a high risk of infection since she was employed on a farm about 20 years before diagnosis. She lived close to several animal species and handled vegetables in inadequate hygienic conditions. Medical and laboratory investigations confirmed the presence of massive echinococcal cystic lesions in each lung and in the liver. Given the peculiarity of the case, pharmacological and surgical treatments were the only conceivable option. The association of pharmacological treatment, surgery, and interventional radiology procedure represented a reliable and effective way to handle a complex case of human hydatidosis. A multi-disciplinary approach was mandatory, resulting in a clear and conclusive diagnosis of CE caused by the zoonotic parasite *E. granulosus sensu stricto* of the G1 genotype.

## 1. Introduction

*Echinococcus granulosus sensu lato* (*s.l.*) is a small tapeworm parasite belonging to the class Cestoda and family Taeniidae [1,2]. The most prevalent species of medical importance infect humans [3,4].

*E. granulosus s.l.* has a worldwide distribution, and the burden of infection in humans and animals is relevant mainly in endemic areas and associated with pastoral communities [5]. The main important factor causing cystic echinococcosis’s (CE) spread is the close coexistence between sheep and dogs raised in inadequate hygienic conditions [6]. Evidence shows CE is highly endemic in Mediterranean countries [7]. In particular, the highest incidence of CE was described in Sardinia and Sicily, corresponding to 6.8/10^5^ and 4.0/10^5^, respectively [8].

The *E. granulosus s.l*. life cycle involves definitive hosts (canids) and intermediate hosts (ruminants) [1]. Humans can accidentally gain the parasite by ingesting contaminated food. The infection, into the circulatory and lymphatic systems, can primarily reach the liver (70–85%) and the lungs (15–47%) [8]. However, the parasite could escape these filters and reach other systems, such as the spleen (0.9–8%), kidney (2–4%) [9], brain (1%), pancreas (0.14–2%), and rarely others [10,11]. Echinococcal cysts are often detected as occasional findings since most subjects have no symptoms or may be silent for years and manifest clinical signs if the lesion grows and compresses surrounding anatomical structures. Subjects with liver involvement usually declare abdominal symptoms, pain, and nausea and may present hepatomegaly and jaundice. Patients with an echinococcal cyst in the lung, such as chest pain, cough, and dyspnea, could report different discomfort. If the cystic lesion determines complications to surrounding vital organs or tissue due to compressive effect, the patients must remove the cyst by operation. In some cases, its rupture may also lead to unpleasant consequences [12].

CE diagnosis in humans is still a challenge and based on imaging techniques: ultrasound (specific for abdominal involvement) and/or conventional chest radiography (useful for thoracic lesions), while computed tomography (CT) and magnetic resonance (MRI) provide more detailed information of the internal part and on vascularization and connection to surrounding tissues, hence, are helpful for surgical intervention or doubt cases [13]. The unclear diagnosis of CE can also be supported by immunological tests (ELISA, immunoblotting (IB), etc.) that detect IgG antibodies against *E. granulosus* and *E. multilocularis* [14,15]. Finally, for patients whose clinical evaluation and serology fail to provide a conclusive response, the direct examination of the cyst and molecular biology analysis after surgical intervention is usually definitive [14,16].

According to World Health Organization (WHO)—Informal Working Group (IWGE) on Echinococcosis [17,18], CE hepatic cysts were classified based on ultrasound evidence as “active”, with two viable substages, the unilocular (CE1) and the multi-vesicular (CE2) with daughter vesicles, the “transitional” with a detachment of endocyst (CE3a) and mainly solid cysts with daughter vesicles (CE3b), then the “inactive”, non-viable usually solid and calcified (CE4 and CE5).

A correct and early diagnosis, along with stadium and localization of the lesion, allows the clinician to choose the appropriate management and the patient’s follow-up correctly [17,19]. Treatment, such as the pharmacological and/or watch-and-wait approach, might be less invasive. In contrast, percutaneous aspiration-instillation and re-aspiration (PAIR) or surgical treatment may be performed only when necessary [19].

Prevention of the spread of parasite in humans and animals is achievable through the elimination of risk factors, such as inadequate health education, extensive farming, clandestine slaughtering, dogs fed with raw viscera and not treated with anthelminthic, vegetables not adequately washed and contact with potentially contaminated soil [6,20,21].

Herein, we report an uncommon clinical case of an adult woman employed as a farmer about 20 years before diagnosis, who was referred to the hospital with severe symptoms attributable to CE. Medical and laboratory investigations confirmed the presence of massive echinococcal cystic lesions in each lung and in the liver. Given the peculiarity of the case, pharmacological and surgical treatments were the only conceivable option.

## 2. Case Description

A 43-year-old woman presenting severe symptoms, including chest pain, dyspnea, coughing, vomiting, and fever, was referred to the emergency department. She complained of progressive weight loss, intermittent fever, and abdominal distension occurring in the previous two years. In addition, the patient reported suffering from allergy attacks with chest pain, dyspnea, and cough for several years. The real-time (RT) polymerase chain reaction (PCR) test was negative for SARS-CoV-2 and performed as per our institute’s mandatory inpatient screening policy during the COVID-19 pandemic.

The patient completed a short questionnaire reporting general data (name, address, date of birth, country of residence for the past 20 years) and information about possible risk factors (occupation, urban or rural residence, contact with animals). The patient’s responses showed that she was born in Romania and moved to Sardinia (Italy) 23 years before her diagnosis. As her first employment, she worked closely with animals on a farm, such as dogs and sheep. She also used to eat unwashed and unrubbed vegetables. A multi-disciplinary evaluation was conducted by an infectivologist, a radiologist, an abdominal surgeon, and an anesthesiologist for an appropriate decision-making approach.

The general physical examination on admission revealed a body temperature of 36.7 °C, a respiratory rate of 21 bpm, a heart rate of 129 bpm, blood pressure of 120/70 mmHg, and oxygen saturation of 92% in room air. Analysis of blood samples showed leukocytosis (11.520 WBCs per microliter), neutropenia (3190 neutrophils per microliter; 28.8%), eosinophilia (5930 eosinophils per microliter; 53.3%), and elevated high-sensitive C-reactive protein (18.45 mg/dL). Moreover, since methicillin-resistant *Staphylococcus aureus*-positive blood cultures were detected, broad-spectrum antibiotic therapy was required. Due to the strong suspicion of parasitic lesions compatible with an echinococcal cyst, determined by several investigations, the patient was initially treated with albendazole (ABZ) (400 mg bis-die). However, the treatment was interrupted after 1 week due to side effects and replaced with mebendazole (MBZ) (100 mg thrice daily).

According to chest radiography, a right chest tube was promptly placed due to evidence of a supposed massive pneumothorax with an ipsilateral mediastinal shift and elevation of the left hemidiaphragm. Purulent, foul-smelling fluid was collected concurrently with continuous air leaks from the right intercostal drainage.

CT examination of the thorax revealed two bilateral voluminous lung cysts, coherent with cystic lung echinococcosis (LCE). The right one was ruptured into the pleural cavity with signs of empyema. Conversely, the left one was preserved in the lung and measured about 15 × 10 × 11 cm, but the water-lily sign was also present, confirming the trend of in-bronchial rupture.

**Figure 1:**
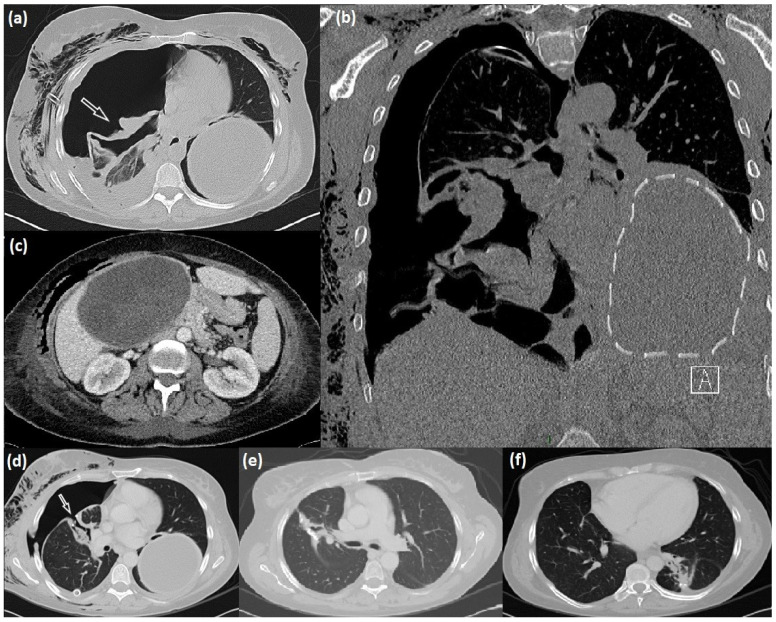
Radiological manifestation of lung-liver hydatidosis. (**a**–**c**) CT scan appearance of the right lung ruptured cyst (arrow), intact giant left lung cyst (dotted line), and liver cyst; (**d**) right bronchopleural fistula (arrow) after cystectomy, and after its surgical repair (**e**); postoperative results after left cyst surgical excision (**f**).

The abdominal ultrasound (US) and CT completion exam revealed an additional giant cyst in the liver with a maximum size of 16 cm (Figure 1).

Clinical and radiological investigations performed via chest radiography and TC (Figure 1) showed two cystic lesions with the typical feature of echinococcal cysts in both lungs and a third one in the liver. According to the WHO, the hepatic cyst was staged as CE3a via US examination.

Serum sample of the patient was analyzed by ELISA Echinococcus IgG kit (DRG, Instruments GmbH, Marburg, Germany), routinely used for screening in our laboratory, and Echinococcus Western Blot IgG (IB) (LDBIO, Diagnostics, Lyons, France), habitually used to confirm the analysis. Antibodies against *Echinococcus* spp. were presented in the routinary and confirmatory analyses, respectively, ELISA and IB.

Given the worsening symptomatology due to the septic scenario sustained by empyema in the right pleural cavity, the patient was referred to a tertiary thoracic surgery department for management.

**Figure 2:**
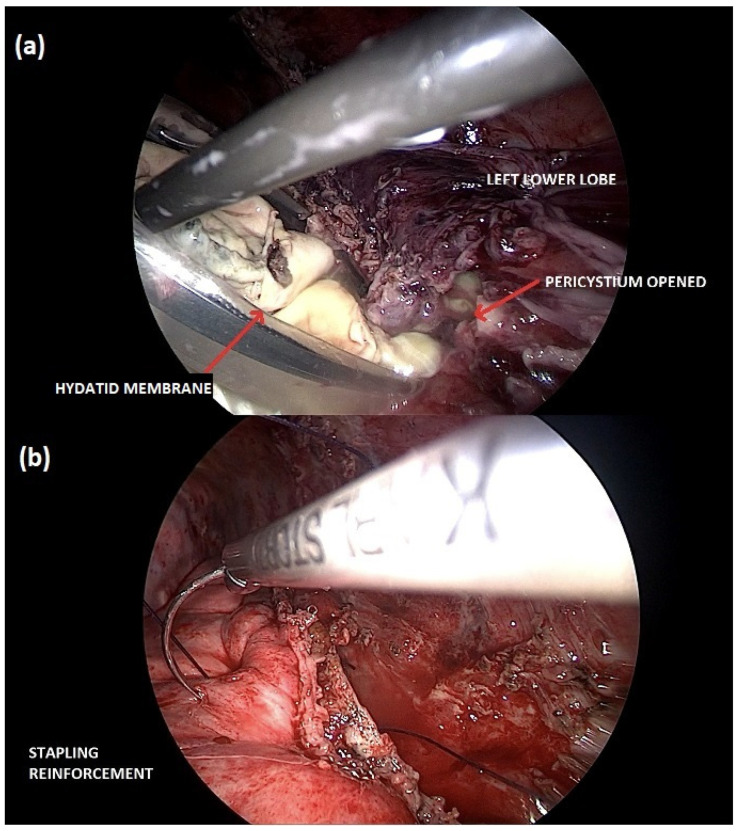
Minimally invasive cystectomy of the left lung hydatid cyst. (**a**) The hydatid membrane is carefully collected into an endo-bag for its retrieval; (**b**) preserving the lung parenchyma, the stapling line is reinforced with a continuous thoracoscopic suture.

A right thoracotomy surgery, with video-thoracoscopic assistance, was performed to remove the cyst, reduce the infectious burden, and perform a pleural debridement. Despite clinical improvement, a redo-surgery was necessary to repair a bronchopleural fistulation one week later. After completely recovering the right pleuropulmonary side, the hydatid cyst on the left lung, which also ruptured into bronchioles, was treated surgically using a total minimally invasive technique (Figure 2).

The patient was discharged on postoperative day 23 after the first operation.

Considering the size and location of the hepatic cyst, the patient was not deemed amenable to surgical resection. Three weeks later, she was referred to the interventional radiology unit for a PAIR ultrasound-guided needle alcoholization of the liver cyst. The postoperative course was uneventful, and investigations at one week and one month showed a complete recovery without signs of local recurrence.

**Figure 3:**
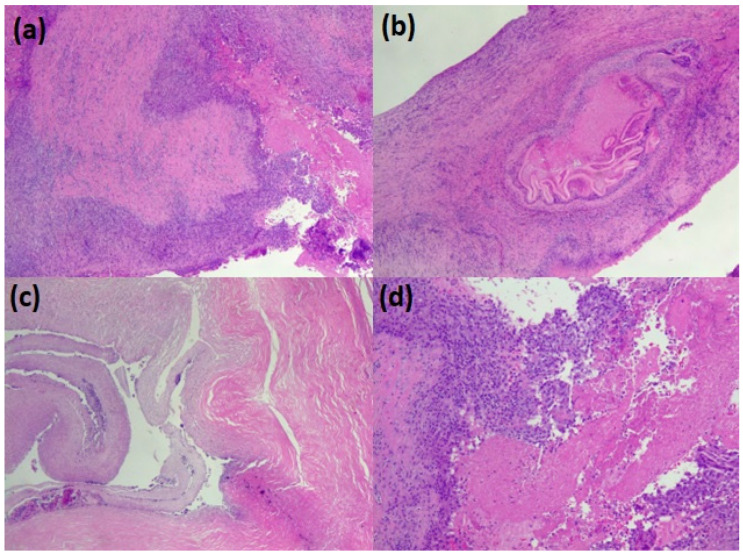
The pathological appearance of hydatid lung parasitosis. (**a**–**c**) the wall of the cyst (the pericyst) with the accompanying chronic inflammation and, in the lumen, some scolean-like parasite residue (**d**).

The parasitic material collected from the lungs was promptly fixed in 10% neutral buffered formalin and embedded in paraffin. Glass slides were prepared by serially cutting the paraffin block into several sections of 3–4 µm and stained with hematoxylin and eosin. Using an optical microscope, histological preparations were observed at 4×, 10×, and 40× to describe the typical parasite features of the parasite: three distinct layers: thick, acellular, and laminated; cellular germinal; brood capsules or protoscolices; and a host-produced granulomatous reaction surrounding the cyst (Figure 3).

Histopathological analysis (Figure 3) of the paraffin section reported a parasitic structure compatible with *E. granulosus s.l.*, and the molecular biology (Table 1) performed via RT-PCR evidenced that the DNA material belonged to *E. granulosus sensu stricto* (*s.s.*), G1 genotype.

Genomic DNA was extracted from the lung lesion by QIAamp DNA FFPE Tissue Kit (Qiagen, Hilden, Germany), specific for purifying DNA from formalin-fixed, paraffin-embedded tissue sections. The NanoPhotometer^®^ N120 (Implen GmbH, Munich, Germany) determined DNA concentration and integrity according to the manufacturer’s instructions.

Subsequently, an RT-PCR [22] was performed on three different single nucleotide polymorphisms (SNPs) (Table 1) for the determination of *E. granulosus sensu stricto* (*s.s.*) and the distinction between G1 and G3 genotypes.

## 3. Discussion

CE represents a critical health problem in many countries worldwide, persisting in many endemic regions and re-emerging in others [5,23]. In particular, in southern Italy, such as the islands of Sardinia and Sicily, sheep and dogs are managed without optimal hygienic conditions, leading to the spread of CE [8].

In the present report, we described a clinical case of an adult female, previously employed on a farm, who was referred to the emergency department with severe symptoms mainly related to the presence of two giant cysts in the lower lung lobes. Our investigations also evidenced a large hepatic asymptomatic lesion.

The clinical manifestations of CE depend on several factors, such as the organ involved, the size of the cyst, and any possible secondary complications that arise [24]. The patient firstly reported several symptoms wrongly related to allergy attacks (chest pain, dyspnea, and coughing), identical to those related to LCE. A differential diagnosis must often be made with diseases, even for mycoses, benign cysts, abscesses, malignant or benign tumors, and caviar tuberculosis [25]. The lung is the second most frequently affected organ [25], mainly in the lower lobe, and patients with LCE may present respiratory symptoms, including a productive or dry cough, hydatic vomica, dyspnea, hemoptysis, chest pain, and fever [26].

However, the clinical presentations depend on whether the cyst is simple or complex. We consider complex LCEs, ruptured lung cysts, even if a secondary infection has not occurred [27]. LCE rupture may occur in the bronchus and/or pleural cavity, with the possibility of developing pleural effusion, pleural CE, allergic symptoms, and anaphylaxis, and it spreading to other lobes through the bronchioles [28]. 

A severe clinical manifestation is not very common in humans. Still, it has been seen rather frequently in other intermediate hosts (ovine, bovine, swine, etc.) with higher risk exposure and environmental pressure. Our case presented two complicated LCEs, with the right one aggravated by fluid leaking into the pleural cavity, requiring immediate surgery. Moreover, the patient presented a ruptured LCE of the bronchi with significant complications, such as an intense cough, chest pain, fever, and emaciation. As reported in the literature, cyst communication into the pleural or pericardial cavity represents a life-threatening condition that can lead to bronchopleural fistulization, hypertensive pneumothorax, pachypleuritis, lung collapse, large residual cavities, and empyema.

Although chest radiography is the first step in examining LCE, other investigations, such as CT scans, are needed to confirm human CE. Serological tests are helpful and reliable tools to support clinical evaluation and corroborate positivity for the patient’s CE. ELISA and Western blotting on the patient’s serum could detect the specific parasite circulating antibodies. Both techniques are mainly used due to their high sensitivity and specificity [13,14,15].

Regarding complete blood count (CBC) parameters, eosinophilia is not a typical finding. It may occur in up to 20% of cases, although it is more frequent in patients with cyst rupture [12]. Peripheral blood eosinophilia was 53.3% in our patient.

CE diagnosis could be made directly on the parasitic material. Cyst fluid aspiration is a minimally invasive technique for specimen retrieval [24]. However, this method must be performed with caution because the leakage of hydatid fluid containing protoscolices may result in reinfection. Otherwise, cystectomy presents fewer complications but is more invasive and must be indicated only when necessary. Regarding our patient, surgery was considered the primary method for LCE as a life-saving treatment. In addition to surgery, chemotherapy represents an adjunctive treatment. The surgical removal of giant intact cysts can be performed after a pre-surgical aspiration to allow for a minimally invasive approach and minimize the risks of intraoperative spreading in the pleural cavity [29]. In our case, up-front surgery was the choice of treatment for both of the LCEs because of cyst rupture, confirmed radiologically and clinically via the recurrences of hydatid vomica.

Uncomplicated CE1 and CE3a cysts can be treated with PAIR depending on their size and location, or when clinical conditions do not allow for a surgical approach. Puncture, aspiration, topical instillation of a scolicide, and re-aspiration have been recommended as first-line therapeutic options for simple CE1 cysts. Both 98% ethanol and 20% saline are the most used scolicides [17,30,31]. The risk of anaphylaxis during a liver CE cyst puncture under ABZ therapy appears to be comparable to surgery [32,33,34,35]. In our case, there were no intraoperative or peri-operative complications following a PAIR liver CE procedure. Conservative benzimidazole therapy is the best option for treating small cysts less than 5 cm in diameter in stages CE1 and CE3a or when the CE already affects several organs [17,30,31]. If ABZ is not tolerated, MBZ can be given instead, as in our patient [31].

Histopathological and molecular biology analyses have a crucial role in CE diagnosis, since they are performed directly on the parasitic material and, consequently, are very useful if other diagnostic tools fail [14,16]. Furthermore, the identification of the *E. granulosus s.s.* species and G1 genotype performed via RT-PCR give important epidemiological information. As broadly reported, G1 is the most represented genotype of intermediate hosts in the Sardinian hyperendemic area [22] and in other Mediterranean and European countries, comprising Romania [36,37], as well as the majority of countries worldwide [38].

Several risk factors were evidenced according to the questionnaire submitted by the patient. In particular, contact with animals, such as widely reported dogs and sheep, is fundamental in perpetuating the biological cycle of the parasite. If their management is inappropriate, the infection could spread into an environment with the dog’s feces. Consequently, any infection can be avoided by washing vegetables properly before consumption [6,39].

Rising temperatures and subsequent heat waves due to ongoing climate change are essential exposure factors when considering social lifestyle modifications and interpenetration with the animal sphere. The Geographical Information System (GIS) and other technologies could improve patient and environmental risk assessment in zonal and interzonal CE by interlacing history in animal and environmental spheres [40]. Thus, it would be interesting to conduct future studies utilizing a One Health approach by using new and innovative field support technologies, such as GIS or other technologies, to better contextualize the case report, the risk assessment context, and the environmental sphere, to be cross-referenced along with the animal and environmental fields.

The farm in which the woman was employed is located in Fordongianus, Sardinia, Italy (39°59′41.55″ N 8°48′34.16″ E).

## 4. Conclusions

Although pulmonary echinococcosis may have benign manifestations, it is a severe disease that should lead to serious complications and critical clinical conditions. Surgical removal of pulmonary cysts is still considered to be the treatment of choice. However, non-invasive or less invasive methods, such as oral chemotherapy, should be incorporated when the patient is unfit for surgery or the perioperative risks are too demanding. Due to the complexity and high-risk scenario, a multi-disciplinary approach was mandatory in the present case. This approach led to a clear and conclusive diagnosis of CE caused by the zoonotic parasite *E. granulosus s.s.* of the G1 genotype.

The association of pharmacological treatment, surgery, and interventional radiology procedure represented a reliable and effective way to handle a challenging human hydatidosis case.

## Figures and Tables

**Table 1:** Real-time PCR for 758, 1123, and 1380 SNPs and their probes. SNP: single nucleotide polymorphisms.

SNPs	Primers	Sequences
758	SNP_758_F SNP_758_R Probe_G1 Probe_G3	5′-GGTTTATGTTGTTGAAGTTGATTGTTTTGT-3′ 5′-AAAACCTAACAACACCTAAATACTCTCAAAGAA-3′ VIC-5′-TGTTGGTATGTAGTGGTGAT-3′ FAM-5′-TGTTGGTATGTACTGGTGAT-3′
1123	SNP_1123_F SNP_1123_R Probe_G1 Probe_G3	5′-CTGGTGTTTGGTTTGTTATGCGTTA-3′ 5′-CCAGTAATAAAAACCGTCAACAAAAGCA-3′ VIC-5′-CGACCTACCAAAATG-3′ FAM-5′-CCGACCTACTAAAATG-3′
1380	SNP_1380_F SNP_1380_R Probe_G1 Probe_G3	5′-GTGATGTGATGAGCGGTAGGG-3′ 5′-CACGACCCATACAAAACAGACCTAT-3′ VIC-5′-CAGGCTAGGAATTGT-3′ FAM-5′-CAGGCTAGAAATTGT-3′

## Data Availability

No new data were created or analyzed in this study. Data sharing is not applicable to this article.
